# Hunting human disease genes: lessons from the past, challenges for the future

**DOI:** 10.1007/s00439-013-1286-3

**Published:** 2013-03-17

**Authors:** Liam R. Brunham, Michael R. Hayden

**Affiliations:** 1 Department of Medicine, Centre for Molecular Medicine and Therapeutics, Child and Family Research Institute, University of British Columbia, Vancouver, Canada; 2 Department of Medical Genetics, Centre for Molecular Medicine and Therapeutics, Child and Family Research Institute, University of British Columbia, Vancouver, Canada; 3 Translational Laboratory for Genetic Medicine, National University of Singapore and the Association for Science, Technology and Research (A*STAR), Singapore, Singapore

## Abstract

The concept that a specific alteration in an individual’s DNA can result in disease is central to our notion of molecular medicine. The molecular basis of more than 3,500 Mendelian disorders has now been identified. In contrast, the identification of genes for common disease has been much more challenging. We discuss historical and contemporary approaches to disease gene identification, focusing on novel opportunities such as the use of population extremes and the identification of rare variants. While our ability to sequence DNA has advanced dramatically, assigning function to a given sequence change remains a major challenge, highlighting the need for both bioinformatics and functional approaches to appropriately interpret these data. We review progress in mapping and identifying human disease genes and discuss future challenges and opportunities for the field.

## Introduction

A principal aim of medical genetics is the identification of the specific genes that, when altered, result in human disease. Most of the success in this endeavor has occurred in the context of Mendelian disorders—genetic diseases thought to reflect the action of a single gene-product with major effect. This group of disorders are recognizable in families by their adherence to one of the canonical patterns of inheritance first described by Gregor Mendel in 1865 and re-discovered in the early 1900s: autosomal recessive, autosomal dominant, co-dominant or sex-linked. Mendelian disorders are individually rare and affect less than 5 % of the population but are extraordinarily numerous. More than 7,000 such disorders have been described (Online Mendelian Inheritance in Man 2012), and countless other “private syndromes” may exist in only small numbers of individuals or even single families.

**Fig. 1:**
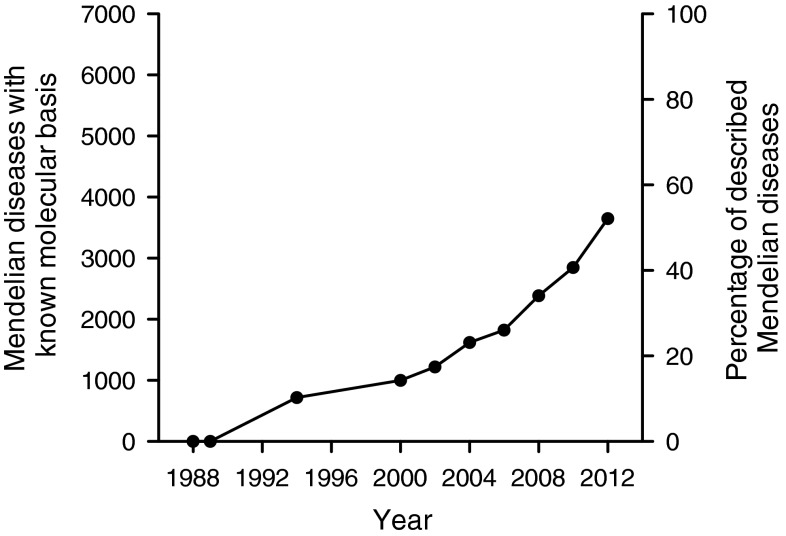
Mendelian diseases of known molecular basis. The *x*-axis shows the time in years from 1988 to present. The left-hand *y*-axis indicates the cumulative number of Mendelian diseases for which a molecular basis is identified and the right-hand *y*-axis expresses this as a percentage of the approximately 7,000 Mendelian disorders that have been described. The first disease Mendelian disease gene to be cloned was the CFTR transporter involved in cystic fibrosis in 1989. Following the release of the first draft of the human genome sequence in 2001, the rate of discovery of Mendelian disease genes increased dramatically. As of November 2012, the molecular basis of 3,650 Mendelian diseases, or slightly more than 50 % of all Mendelian diseases, is known. Data are adapted from (McKusick 2007; Antonarakis and Beckmann 2006; Hamosh et al. 2005; Pearson et al. 1994; Online Mendelian Inheritance in Man 2012)

More than 3,600 Mendelian disorders, or ~50 % of all those described, have now been associated with a specific molecular defect (Online Mendelian Inheritance in Man 2012) (Fig. 1), speaking to both the remarkable success and the ongoing opportunity that exists in human disease gene mapping. Pathogenic mutations have been described in slightly more than 5,000 human genes (Human Gene Mutation Database. http://www.hgmd.cf.ac.uk/ac/hahaha.php). However, data from other organisms suggest that a much higher percentage of the ~22,000 human genes may be associated with phenotypes when altered. For example, systematic gene mutation screens in yeast and mice suggest that most of the genes in those organisms are non-lethal and associated with a discernible phenotype when disrupted (Ayadi et al. 2012; Winzeler et al. 1999; Hillenmeyer et al. 2008).

## Mapping genes by positional cloning

For most of the modern era of human genetics, the principal method for the identification of disease-associated genes was positional cloning using linkage analysis. In this methodology, one or more pedigrees in which the trait of interest is observed to segregate are used for study. DNA from both affected and unaffected individuals are genotyped for polymorphic markers spread throughout the genome. Making use of the recombination that occurs in meiosis, one can then identify a chromosomal region that, based on the presumed mode of inheritance, shows segregation of a disease-associated haplotype in affected individuals, and of a non-disease-associated haplotype in unaffected individuals.

Linkage analysis was first described in fruit flies 100 years ago (Sturtevant 1913) but it was not until the discovery of naturally occurring polymorphic DNA markers in the 1980s that this tool first became available to map human disease genes (Botstein et al. 1980). One of the early successes was mapping of the gene for Huntington disease in 1983 (Gusella et al. 1983). Other examples that soon followed included the genes for cystic fibrosis (Rommens et al. 1989; Knowlton et al. 1985; White et al. 1985), Duchenne muscular dystrophy (Koenig et al. 1987) and many others.

To proceed from an area of linkage to a disease-associated mutation requires knowledge of the specific genes that exist in that chromosomal region. In the pre-human genome era, this involved examination of additional recombinant chromosomes to further refine the linkage interval and a variety of molecular biology techniques to identify expressed genes in the region and search for mutations. The Herculean nature of this task is demonstrated by the fact that it took international research groups a decade to go from identifying the Huntington disease locus to cloning the disease gene and identifying pathogenic mutations, in that case a triplet expansion in the *HTT* gene (MacDonald et al. 1993). With the completion of the initial sequencing of the human genome (Lander et al. 2001; Venter et al. 2001), this task has been tremendously facilitated with a corresponding acceleration in the identification of Mendelian disease genes (Fig. 1). From the 1980s to the year 2000, the molecular basis of approximately 1,000 Mendelian disorders had been discovered. In the first 10 years following the publication of the human genome, this number more than tripled to over 3,000, representing one of the indisputable fruits of the Human Genome Project with direct impact to the patients affected by these disorders and their families.

A form of linkage analysis that can be used in consanguineous families with suspected autosomal recessive traits is homozygosity mapping. This makes use of the principle that DNA markers in the chromosomal region immediately adjacent to the disease locus should be homozygous by descent in such cases (Lander and Botstein 1987). This strategy can efficiently identify genomic regions in which candidate genes can then be tested for the presence of pathogenic mutations. Homozygosity mapping has recently been used in combination with high-density whole genome genotyping to identify disease genes in patients in whom homozygosity by descent is suspected (Molho-Pessach et al. 2012).

The biological and medical importance of these disease-gene discoveries cannot be overstated. Once a disease gene for a Mendelian disorder is identified, an enormous amount of information about the biological function of that gene is provided by the phenotype of individuals in whom it is dysfunctional. Conversely, study of the biological pathways that the gene-product is involved in illuminates disease pathophysiology. From a clinical perspective, identification of a disease gene opens the door to diagnostic and predictive testing where appropriate. For instance, predictive testing for Huntington disease became available in Canada in 1987 using linkage analysis based on localization of the Huntington disease gene (Fox et al. 1989).

Moreover, mutation identification can directly lead to therapeutic insight and already significant advances in targeted therapies have been achieved by understanding genotype. For instance, cystic fibrosis (CF) is caused by a variety of different mutations in the chloride transporter encoded by the *CFTR* gene. Some mutations, such as the common **Δ**F508 mutation, result in inability of the encoded protein to reach the plasma membrane. Other mutations, such as G551D which is present in ~5 % of patients, are associated with transport of the protein to the cell surface but failure of the channel to open. Ivacaftor is a novel small molecule that potentiates the *CFTR* channel at the cell surface (Eckford et al. 2012) and leads to significant improvements in lung-function in patients with the G551D mutation (Ramsey et al. 2011). In January 2012, this drug was approved by the Food and Drug Administration for treatment of CF in patients who carry this mutation, making this the first genotype-specific therapy for CF.

A second example involves lipoprotein lipase deficiency, a condition characterized by extremely high levels of triglycerides in plasma and recurrent attacks of painful pancreatitis. Identification of the *LPL* S447X mutation and understanding of its biochemical phenotype as a gain-of-function variant (Ross et al. 2005) ultimately led to the development of the first approved gene therapy product for humans (Yla-Herttuala 2012). Both of these examples illustrate how the understanding of genotype can lead to profound clinical insight, both by guiding appropriate patient selection and by directly leading to the development of new therapeutic products.

## Role of next-generation sequencing in disease gene identification

The development of massively paralleled (next generation) sequencing has lead to dramatic acceleration in the pace of genetic discovery. These technologies have enabled two major advances of relevance to the discovery of disease-associated genes. The first is the ability to readily sequence the genome of a single person, thus allowing identification of mutations specific to that individual (previous “human genomes” represented consensus sequences of DNA from several individuals and were, therefore, not suited to the identification of rare mutations). The second major application of next-generation sequencing is the ability to perform whole exome sequencing (WES) in which a targeted capture strategy is used to sub-select the protein-coding exonic portion of the genome and generate sequence data of all known genes (Teer and Mullikin 2010). This results in sequence data covering ~30 MB of the genome, or ~1 % of that examined by whole genome sequencing (WGS), enabling the identification of mutations that result in changes to the amino-acid sequence of encoded proteins while substantially reducing the computational requirements associated with analyzing the resulting data. Examining only the exonic portion of the genome is justified on the basis that the vast majority of Mendelian disease-associated mutations identified by positional cloning strategies result in disruption of the protein-coding sequence (Stenson et al. 2009).

Sequencing of human exomes was first reported in 2009 (Ng et al. 2009) and the use of this technology to discover the genetic cause of a Mendelian disorder, Miller syndrome, followed soon after (Ng et al. 2010). Importantly, this demonstrated that WES makes tractable those conditions that are too rare and in which appropriately sized families are not available for positional cloning strategies, illustrating the power of this approach in situations where only small numbers of affected individuals are available for study.

WES and WGS strategies have now been used to elucidate the molecular etiology of an ever-expanding list of Mendelian disorders, as reviewed elsewhere (Gonzaga-Jauregui et al. 2012; Ku et al. 2011; Bamshad et al. 2011). While this undoubtedly represents a major advance for mapping human disease genes, several challenges remain. Capture methods remain imperfect and can result in unequal depth of coverage at different exonic regions. Our incomplete annotation of all human genes results in a necessarily incomplete view of the human exome. The analytical challenges are also significant. Each sequenced exome results in 20,000–25,000 variants relative to the reference sequence (Bamshad et al. 2011). How do we go from this huge number of variants to a single pathogenic mutation? Typically a number of filtering steps are employed (Bamshad et al. 2011), for instance, by ruling out any variants found in public databases such as dbSNP, 1000 Genomes Project, HapMap or locally available exome databases (Stitziel et al. 2011). This rests on the assumption that the causative variant will be extremely rare and that any individual with the variant will be affected (i.e., the variant is fully penetrant). While this is an efficient method to rule out the vast majority of variants, and is reasonable in scenarios where the disease-causing variant is hypothesized to be novel, it may be overly restrictive. As more genome and exome sequences are deposited in public repositories, the assumption that any variant found in these databases can be ruled out as disease-causing becomes increasingly difficult to justify. This is especially the case for recessive conditions in which the carrier state is relatively common (e.g., *HFE* gene mutations associated with hereditary hemochromatosis) or in situations in which the causative mutation is not fully penetrant. As a result, many groups now set frequency-based tolerances for variants found in dbSNP or other databases, such that variants present in fewer than 1 % of chromosomes are carried forward as candidates for a rare pathogenic mutation (McDonald et al. 2012).

**Fig. 2:**
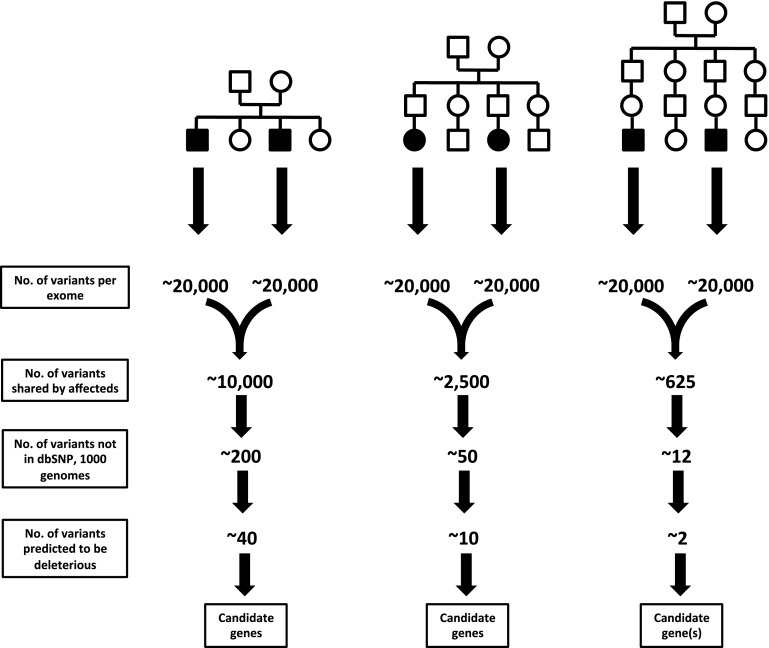
Theoretical example of filtering steps used to limit number of variants identified by exome sequencing. The three examples indicate two affected individuals who are related to different degrees. Comparing the exomes of siblings, 1st cousins or 2nd cousins will limit candidate variants to the roughly 1/2th, 1/8th or 1/32nd of the genome, respectively, that is shared by two such individuals. Additional discrete filtering and bioinformatics steps can further reduce the number of candidate variants. Examples assume that all but 2 % of variants will be identified in public databases such as dbSNP and 1000 Genomes Project and that approximately 20 % of novel variants will be predicted to be deleterious to the function of the encoded protein

An additional method to reduce the complexity of exome sequence data is through integration with family data or with traditional linkage analysis. For example, if two affected first cousins are used, the number of variants to be considered as candidates is reduced to the roughly 1/8th of the genome shared by two such individuals (Fig. 2). Similarly, the use of two affected second cousins reduces the number of shared variants to 1/32nd of the genome.

Integration of genome or exome sequencing with linkage analysis is also a powerful approach that can narrow the list of candidate genes. This approach has been successfully used to identify mutations associated with metachondromatosis (Sobreira et al. 2010), osteogenesis imperfect (Volodarsky et al. 2013; Cho et al. 2012), Chartcot-Marie-Tooth disease (Kennerson et al. 2013), spinocerebellar ataxia (Lee et al. 2012), opsismodysplasia (Below et al. 2013), distal arthrogryposis (McMillin et al. 2013), craniocervical dystonia (Charlesworth et al. 2012), dyskinesia and facial myokymia (Chen et al. 2012), thoracic aortic aneurysm syndrome (Boileau et al. 2012), distal hereditary motor neuropathy (Beetz et al. 2012), spinal muscular atrophy (Zhou et al. 2012), familial pityriasis rubra pilaris (Fuchs-Telem et al. 2012) and many others (Ku et al. 2011). In many cases, this approach enables gene mapping in only a single affected individual and can substantially narrow the list of candidate mutations generated by WES/WGS. For instance, in a study of metachondromatosis, the inclusion of linkage data reduced the number of candidate genes with disruptive mutations from 109 across the whole genome to only one found in a linkage region (Sobreira et al. 2010).

These tremendous technological breakthroughs raise the tantalizing possibility that in the near future we may discover the molecular etiology of most, or even all, Mendelian diseases. Achieving this remarkable goal will require world-wide collaboration bringing together clinicians caring for patients with rare Mendelian diseases and experts in genome technologies as well as computational biology. Consortia such as the Finding of Rare Disease Genes (FORGE) in Canada, the International Rare Diseases Research Consortium in Europe and the Centers for Mendelian Genomics by the National Institutes of Health in the United States (Bamshad et al. 2012) should have a major impact on applying genomic technologies to unraveling Mendelian disorders.

## Unexpected phenotypes as a clue to modifier or suppressor mutations

The widespread availability of sequence data also permits identification of unexpected splits between genotype and phenotype that may suggest the presence of a suppressor or modifier mutation. Examples of this include individuals with pathogenic mutations in the LDL receptor gene but normal cholesterol levels (Hobbs et al. 1989), or patients with an expanded CAG repeat in the Huntington disease gene who have not manifest disease by the 95th percentile of age expected for that CAG repeat length, or conversely those that have manifest disease prior to the 5th percentile of age expected (Langbehn et al. 2004; Brinkman et al. 1997). Other examples include individuals homozygous for the ApoE ε4 allele who remain free of Alzheimer’s late into life, or individuals homozygous for null alpha-1 antitrypsin alleles (i.e., ZZ genotype) who do not develop obstructive airways disease. All of these examples point to the presence of a mutation in another gene that masks the expected phenotype.

We recently described two families with mutations in the *SCARB1* gene that encodes the HDL receptor, SR-BI (Brunham et al. 2011) and was recently reported to be a cause of high HDL-C levels in humans (Vergeer et al. 2011). While most individuals who carried the *SCARB1* mutations had elevated HDL cholesterol (HDL-C) (>95th percentile for age and gender), one mutation-carrier was observed to have unexpectedly low HDL-C (15th percentile). Sequencing of candidate low HDL-C genes in this individual led to the identification of novel mutation in *ABCA1*, V2091I, that segregated with low HDL-C and may be the cause of this individual’s low HDL-C thereby explaining the unexpected phenotype in that patient.

As more genome and exome data become available for a larger and larger number of individuals, we will have increasing opportunity to identify unexpected phenotypes in the presence of a given genotype. The importance of these observations is in pointing to the presence of potential suppressor genes that in many instances may represent novel therapeutic targets. For instance, individuals with familial hypercholesterolemia (FH)-causing mutations but normal levels of LDL-C suggest the presence of a mechanism for lowering LDL-C that is independent of the LDL receptor and would, therefore, be effective in individuals with FH, one of the most common causes of inherited high cholesterol. The identification of such suppressor mutations may lead to novel approaches to modify the course of illness by identifying therapeutic targets that have already been validated in relevant human disease models.

## Disease genes for common diseases

In contrast to the remarkable success achieved in the identification of genes for Mendelian diseases, common diseases have proved much more difficult to unravel. Linkage studies for complex disease proved extremely difficult due to a lack of sufficient genomic resolution to identify disease-associated loci using microsatellite markers and inadequate power to detect an association, largely due to the significant locus heterogeneity that characterizes common disease (John et al. 2004; Altmuller et al. 2001; Xiong and Guo 1998). An alternative approach was therefore developed, genetic association studies, in which the frequency of common DNA polymorphisms is compared in unrelated cases versus controls. Association studies provide much greater power to detect variants associated with common disease than does linkage analysis, particularly when the risk conferred by the gene is modest (Risch and Merikangas 1996). The DNA marker studied need not be causal for the disease in question; because of patterns of linkage disequilibrium (LD) in the genome, nearby DNA markers tend to be inherited together. Indeed, the discovery that recombination tends to occur at specific “hot spots” (McVean et al. 2004; Daly et al. 2001) suggested that a single “tag” single nucleotide polymorphism (SNP) could capture most of the common DNA variation in a particular genomic region (Johnson et al. 2001).

Initial association studies were limited to examining candidate genes and therefore were not suited to the identification of novel genetic risk loci (Tabor et al. 2002). Multiple technical and conceptual advances changed this, including the development of high-throughput genotyping technologies capable of genotyping thousands of SNPs in large numbers of individuals, large catalogues of SNPs (Sachidanandam et al. 2001), knowledge of patterns of LD in the genome (International HapMap Consortium 2003) and analytic frameworks to analyze enormous datasets. By 2006, genome-wide association studies (GWAS) became a reality (Hirschhorn and Daly 2005). GWAS involves genotyping high-density panels of polymorphisms in several thousand cases and controls to identify common variants (>1 % allele frequency) that are hypothesized to underlie some portion of the heritability of common diseases. The basis for these studies is the common disease–common variant hypothesis which posits that genetic risk for common diseases will be due at least in part to a small number of disease-predisposing alleles per locus that exist at high frequency (Lander 1996; Chakravarti 1999).

More than 1,000 GWAS have now been reported with evidence of association of thousands of SNPs for dozens of common conditions (Hindorff et al. 2012) and has led to substantial advances in our understanding of the role of common variation in common disease (Altshuler et al. 2008). In general, GWAS has been successful in identifying numerous variants that are associated with common disease at high levels of statistical significance; however, most of these alleles confer only small effects sizes (Odds ratios <1.5). As such, GWAS has largely not succeeded in identifying disease genes with large effects, raising the concept of the “missing heritability” of common disease (Manolio et al. 2009). Rare variants or structural variants not represented on GWAS genotyping panels may be a source of this missing heritability (Manolio et al. 2009). Alternatively, estimates of heritability may themselves be inaccurate, for instance, due to the effect of gene–gene interactions which are generally not incorporated into heritability estimates and would have the effect of increasing the heritability apparently left to account for (Zuk et al. 2012).

How will we move forward to uncover the remaining heritability of these common conditions? One exciting approach is the use of genotyping and next-generation sequencing technologies to identify rare and private variants. The alternative to the common disease–common variant hypothesis is that most variants underlying disease in humans will be individually rare because any variant with a major effect on fitness will tend to be removed from the population by the actions of natural selection, thus keeping the frequency of such variants very low. Indeed, rare variants are 4 times more likely to be functional (based on bioinformatics predictions) than are more common variants (minor allele frequency greater than 0.5 %) (Tennessen et al. 2012). Most of the variation in the human genome consist of common variants that arose prior to the migration of modern humans out of Africa and are shared between continental populations—these are the variants represented in HapMap (International HapMap Consortium 2003) that form the basis for GWAS. However, the vast majority of the coding, polymorphic alleles in humans are individually rare and represent evolutionarily recent mutations that have occurred with the rapid population expansion over the past 10,000 years (Tennessen et al. 2012). If common human diseases are characterized genetically by marked allelic and locus heterogeneity, as has been postulated (McClellan and King 2010), then new methods will be required to identify the rare alleles that contribute to human disease.

A study of sick sinus syndrome (SSS) in the Icelandic population is illustrative of how these rare variants can be identified. These investigators initially performed GWAS in a population of ~800 cases and nearly 40,000 population-based controls to define a region on chromosome 14q11 that contained 3 SNPs associated with SSS at genome-wide levels of significance. Subsequent WGS in a selection of 87 individuals identified 11 million variants that were then imputed into the GWAS cases and controls using long-range haplotype phasing (Kong et al. 2008). This approach identified a rare coding variant (minor allele frequency 0.38 % in Icelandic population) in the *MYH6* gene, located at the 14q11 locus, that was associated with SSS with an Odds ratio of 12.5 and *p* value of 1.5 × 10^−29^ (Holm et al. 2011). This association was further validated by direct genotyping in an additional set of SSS cases and controls. This study provides compelling evidence of a rare variant with large effect size that impacts a complex trait and provides impetus for efforts to identify such variants for common diseases broadly. This study also suggests that combining GWAS, WGS and imputation in a population isolate serves as one method by which this class of variant can be identified.

Imputation of rare variants identified by WGS into patients who have been genotyped for GWAS panels provides a cost-effective method for performing association studies of a large number of rare, potentially functional variants. This approach was used, also in the Icelandic population, to identify a missense substitution (R47H) in the *TREM2* gene, with an allele frequency of 0.12–0.63 % in the various populations studied, that confers a threefold increased risk of Alzheimer’s disease (Jonsson et al. 2012). The same *TREM2* variant was simultaneously identified by a candidate gene sequencing approach and was found to occur significantly more frequently in individuals with Alzheimer’s compared to controls (Guerreiro et al. 2012). The imputation of rare variants provides one attractive means to extend the unbiased nature of GWAS to low-frequency variants selected for potential functionality. Similarly, “exome-chip” approaches will soon enable direct genotyping of several hundred thousand putatively functional low-frequency coding variants identified by WGS or WES (Kathiresan and Srivastava 2012).

Ultimately, the most comprehensive approach for searching for rare variants that influence common disease will be WGS or WES of large numbers of cases and controls to identify variants associated with these phenotypes. Though this strategy remains both technically and economically prohibitive at present, it will no doubt be deployed in the near future. A key consideration of such studies would be the statistical power to detect an association. Because power decreases as a function of allele frequency, very large sample sizes will likely be necessary to identify disease-association of rare variants. With several hundred cases and controls, very few genes have adequate power to identify a rare variant that confers an Odds ratio of 5 or more (Tennessen et al. 2012). For smaller effect sizes, such as 1.5, several thousand cases and controls will be required (Raychaudhuri 2011). Accepted levels of statistical significance that take into account the burden of multiple testing for rare variants across the genome or exome need to be established and will be more stringent than levels used in GWAS reflecting the far greater number of rare than common variants in the genome; a *p* value of 10^−11^ has been proposed (Raychaudhuri 2011). Issues of incomplete penetrance, genetic heterogeneity, and gene–gene and gene-environment interactions will be additional complications requiring close attention.

## Using population extremes to identify disease genes

**Fig. 3:**
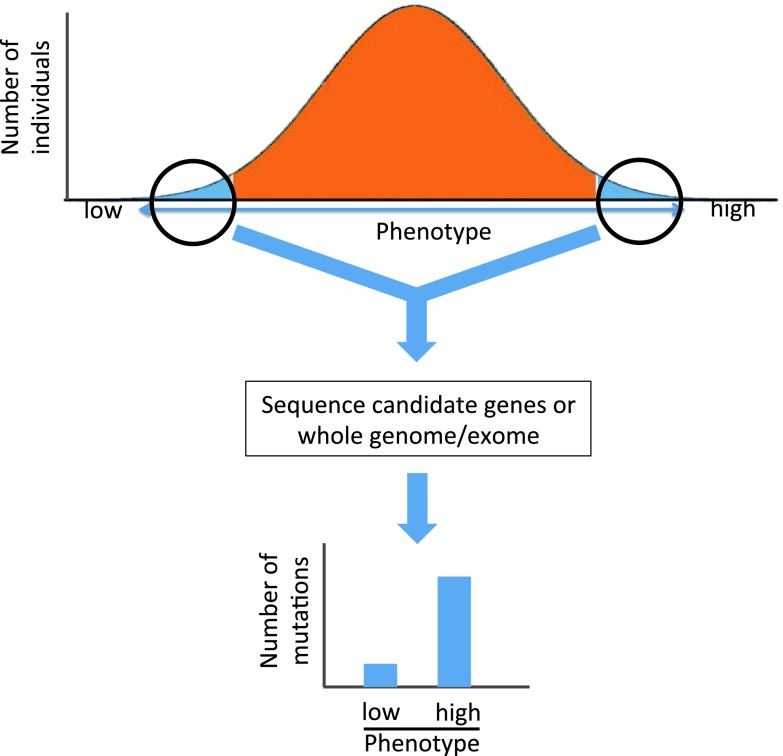
Sequencing the extremes to identify rare variants involved in common disease. The individuals from the high and low ends of the population distribution of a trait are chosen for study. Either candidate genes or whole exomes or genomes are sequenced and the number of rare variants in a given gene is compared between the high and low groups. An excess of rare sequence variants in a given gene in the high versus low (or vice versa) group provides evidence for a role of that gene in the phenotype under study. See text for examples of where this has been used

An additional strategy to identify rare variants that confer disease risk involves studying individuals representing the ends of a quantitative phenotype, a strategy known as “sequencing the extremes” (Fig. 3). The prototypical study to establish this approach involved sequencing 3 candidate genes for low levels of HDL-C, *ABCA1*, *APOA1* and *LCAT*, in individuals with the lowest and highest 5 % of HDL-C levels in a population (Cohen et al. 2004). The number of non-synonymous sequence variants in these genes that were unique to either the low or high HDL-C groups was compared and revealed a statistically significant excess of rare mutations among individuals with low HDL-C (20 versus 3 in the low versus high HDL-C groups). Most of these mutations were in the *ABCA1* gene (Cohen et al. 2004).

This method has subsequently been used to provide evidence that rare variants in specific genes influence levels of LDL-C (Cohen et al. 2005) and intestinal sterol absorption (Cohen et al. 2006; Fahmi et al. 2008), triglycerides (Hobbs et al. 1989; Romeo et al. 2007), body mass (Ahituv et al. 2007) and high levels of HDL-C (Brunham et al. 2011; Tietjen et al. 2012). A re-sequencing study of four genes implicated by GWAS to be associated with hypertriglyceridemia demonstrated that these same genes harbor an excess of rare variants, with 28.1 % of hypertriglyceridemic individuals carrying a rare variant in one of these genes compared to 15.3 % of individuals with normal triglyceride levels (Johansen et al. 2010). Importantly, this demonstrates that some portion of the variability of common disease is likely due to rare variants in the same genes in which common variation has been associated with these traits.

Sequencing the extremes has emerged as a powerful tool to provide evidence for the role of rare mutations in specific genes impacting complex traits. However, a limitation of this approach has been that it is restricted to candidate genes. For instance, in the study of low levels of HDL-C described above, the *ABCA1*, *APOA1* and *LCAT* genes were chosen because mutations in each of these genes are known to cause rare, recessive Mendelian conditions of low HDL-C. To identify novel disease genes, this approach would need to be extended to perform WGS or WES in the population extremes of a trait.

Indeed, such a strategy has recently been reported to discover a gene that impacts *Pseudomonas* infection in individuals with cystic fibrosis (Emond et al. 2012). Most patients with CF become colonized with *Pseudomonas* early in life with a median of 1 year (Li et al. 2005), but substantial variability exists in this phenotype. Emond et al. (2012) identified two groups of patients representing the extremes of *Pseudomonas* colonization: those who became chronically infected prior to the 10th percentile age of onset and those who remained culture negative beyond 14 years of age. Exome sequencing of these individuals identified a single gene, *DCTN4*, that showed statistically significant evidence of association with time to *Pseudomonas* infection after correcting for multiple testing. This study suggests that the extreme phenotype approach can be successfully combined with exome sequencing to identify novel genes involved in complex traits. More studies are clearly needed to determine if this will prove to be a successful approach in general and new analytic methods are required to assess the statistical significance of rare variants identified through such approaches.

## De Novo mutations and copy number variants in human disease

The availability of WGS and WES also affords the opportunity to delineate DNA variants that are present in the genome or exome of an offspring but absent in that individual’s parents. This trio-sequencing approach not only enables accurate determination of the mutation rate but also importantly allows for precise identification of de novo mutations at single nucleotide resolution (Veltman and Brunner 2012). De novo mutations are, in a sense, the most extreme form of rare mutations, in that they may be private and have not been subject to selective pressure in previous generations. These variants are therefore prime suspects for playing a role in human disease.

Several recent studies have examined the contribution of de novo mutations to common disease. Four recent studies reported exome sequencing of ‘trios’ (affected child and parents) or ‘quads’ (affected child, unaffected sibling and parents) in families with autism spectrum disorder (ASD) (Neale et al. 2012; O’Roak et al. 2012; Sanders et al. 2012; Iossifov et al. 2012). These studies found a small increase in de novo mutations in ASD cases compared to unaffected controls that tended to occur in genes that are biologically related to each other or to previously known ASD genes. These mutations were predominantly paternal in origin, and the number of mutations increased with increasing paternal age, consistent with the known increased risk of ASD to children of older fathers.

While the ability to accurately detect such mutations opens the door to many exciting lines of discovery that may account for some of the missing heritability of common disease, these studies also highlight many challenges. For instance, how do we interpret the biological significance of these mutations and link them with disease? In particular, how do we distinguish a single de novo mutation, even an apparently deleterious one, from the substantial background of mutational events that these studies have demonstrated? These studies also highlight the extreme genetic heterogeneity of these disorders. For example, of the more than 120 genes implicated in the studies of autism, only six genes had disruptive mutations in more than one individual (Muers 2012). While this may suggest that ASD represents hundreds of genetically distinct entities, it remains unclear to what extent this sub-classification will yield meaningful clinical insight.

One class of genetic variation with a large contribution from de novo events is structural variation that affects the number of copies of a particular chromosomal region and can thereby impact phenotypes via a gene-dosage effect of the implicated gene or genes. Our understanding of the diversity of copy number variants (CNVs) has advanced dramatically in recent years and we now know that CNVs are remarkably common in the genomes of normal individuals (Sebat et al. 2004; Iafrate et al. 2004). While some CNVs are polymorphic in human populations, most are rare or private. Most CNVs are thought to arise due to non-allelic homologous recombination between highly identical regions of the genome (Girirajan et al. 2011). CNVs affect an order of magnitude more base pairs in the genome than do SNPs (Lupski 2007), and more than one-third of all human genes may be partially or totally in the region of a CNV (Gonzaga-Jauregui et al. 2012). The Database of Genomic Variants currently listed more than 15,000 CNV loci in the human genome (The Database of Genomic Variants 2013).

CNVs have long been associated with ‘genomic disorders’—rare highly penetrant syndromes typically associated with neurodevelopmental delay (Girirajan et al. 2011). Rare CNVs have also been documented to play an important role in psychiatric and behavioral conditions such as schizophrenia and ASD (Xu et al. 2008; Walsh et al. 2008; Sebat et al. 2007). More recently, a CNV at 17p was shown to influence obesity traits in mice, with deletion of this region leading to obesity and metabolic syndrome, and duplication leading to protection from obesity (Lacaria et al. 2012). CNVs have also been reported to play a role in congenital heart disease (Soemedi et al. 2012; Hitz et al. 2012), epilepsy (Helbig et al. 2009) and many other conditions (Zhang et al. 2009; Stankiewicz and Lupski 2010; Girirajan et al. 2011).

Comprehensive detection of CNVs in the human genome remains a challenge but recent technological advances have accelerated our ability to do so. In particular, array comparative genome hybridization and paired-end mapping, in which the presence of a CNV is suggested by a size difference between the fragment length and the corresponding region of the reference sequence (Korbel et al. 2007). Notably, these methods allow for accurate detection of smaller CNVs with more precise resolution of breakpoints. Widespread application of these techniques to an expanding list of common diseases should provide further insight into the role of CNVs in common genetic diseases.

## What constitutes proof that a mutation causes a disease?

How do we prove that a mutation in a gene is causative of disease? Linkage analysis can provide evidence of DNA variants in a gene that segregate with disease in a family. Finding mutations in the same gene in unrelated families provide additional evidence of causality. For highly characteristic rare phenotypes that segregate in a family, the accepted standard is replication across three independent families. For variants identified in extremely rare or private phenotypes, the burden of proof is less well defined and new analytic methods are required. This underscores an important principle relevant to both traditional and contemporary gene mapping. Namely, that finding different, rare, pathogenic mutations in the same gene or in the same biological pathway, in unrelated individuals with the same phenotype provides important support for that gene being involved in a given biological process (McClellan and King 2010).

Bioinformatics algorithms such as Polyphen (Adzhubei et al. 2010), SIFT (Kumar et al. 2009), SNAP (Johnson et al. 2008) or PANTHER (Mi et al. 2010) are frequently used as a first approximation for determining if a given DNA or amino-acid sequence variant is likely to impair the function of the encoded protein. Most of these programs rely on some combination of conservation of the site in question in related proteins within and across species as well as knowledge of structure of the protein. Functional data for a limited number of genes suggest that these methods have reasonable accuracy for the prediction of deleterious effects on specific proteins (Ng and Henikoff 2006). For example, PANTHER correctly identified as deleterious or benign ~95 % of rare variants in the *ABCA1* gene (Brunham et al. 2005). However, none of these methods are perfectly sensitive or specific. Indeed, in the exome sequencing study that identified the molecular cause of Miller syndrome, the use of Polyphen as a filter for functional variants would have excluded the mutation in *DHODH*, ultimately found to be causative of that disease (Ng et al. 2010). Moreover, agreement among the various methods are poor: in a deeply sequenced set of exome data, about half of all detected SNPs were predicted to be functional by at least one of seven different bioinformatics programs, but the various programs were in full agreement on only 1 % of variants (Tennessen et al. 2012).

Functional studies are, therefore, crucial to establish which DNA variants truly impact protein function and are causal in disease. This remains a major rate-limiting step, because, for most gene-products, a readily availably functional assay does not exist. For many genes, we simply do not know enough about the function of the gene-product and therefore lack methods for testing the consequence of sequence variation, especially in high-throughput fashion. Full functional annotation of all human genes remains a lofty and distant goal.

In GWAS, proof generally takes the form of a level of statistical significance. In particular, a *p* value threshold of 5 × 10^−8^ is felt to reflect the nominal significance threshold of a finding being due to chance 1 in 20 times corrected for the approximately 1 million independent tests incurred in a genome-wide scan (Pe’er et al. 2008). However, it is important to note that this provides evidence only of association and not of causation. Despite these high levels of statistical stringency, most variants identified by GWAS are in non-coding regions of the genome and the causal variant is often unknown. This may be explained by linkage disequilibrium between the associated variant and a true functional variant, or by a regulatory effect of a non-coding variant. Differentiating between these possibilities can be extremely challenging.

For example, a locus at 1p13 was known to be strongly associated with LDL cholesterol in a meta-analysis of >100,000 individuals (Teslovich et al. 2010). Fine-mapping of this region identified 6 SNPs that showed the greatest degree of association in the genomic region between the genes *CELSR2* and *PSRC1* (Musunuru et al. 2010) but the causative variant was not obvious. Sequential testing of these variants identified a single SNP that resulted in increased gene expression by creating a transcription factor binding site for *SORT1*, a gene shown to regulate hepatic VLDL secretion.

A second example involves variants in a gene desert at chromosome region 9p21 associated with coronary artery disease (McPherson et al. 2007). Through a series of computational and experimental approaches using immortalized cell lines, Harismendy et al. 2011 demonstrated that a single SNP in this region interrupts a binding site for *STAT1*, a transcription factor implicated in inflammatory responses, and that this locus participates in a long-range physical interaction with the *CDKN2A/B* locus. These two examples highlight how challenging it is to move from genetic association to mechanistic insight, but provide important models for how this may be achieved.

## Conclusions

The past decade has witnessed tremendous progress in the identification of the molecular bases of human disease. Indeed, for Mendelian disorders, it is conceivable that in the future we may possess a complete catalogue of the genetic basis for each of these conditions. Common diseases continue to be much more challenging to address, but recent advances in sequencing and genotyping methods are yielding exciting results. Next-generation sequencing has dramatically altered our ability to identify human disease genes, but many challenges remain. In particular, our ability to ascribe functional significance to a given DNA variant remains limited. Ultimately, the identification of the molecular causes of disease will continue to illuminate the pathophysiology of both common and rare conditions and offer opportunities for improvements in the diagnosis, treatment and prevention of disease.
